# Evaluation of clinical trials of ethnomedicine used for the treatment of diabetes: A systematic review

**DOI:** 10.3389/fphar.2023.1176618

**Published:** 2023-04-07

**Authors:** Gul Rehman Elmi, Kamil Anum, Kalsoom Saleem, Rameesha Fareed, Sobia Noreen, Haiyan Wei, Yongxing Chen, Avirup Chakraborty, Masood Ur Rehman, Shi Liyuan, Muhammad Abbas, Yongtao Duan

**Affiliations:** ^1^ Henan Provincial Key Laboratory of Pediatric Hematology, Children’s Genetics and Metabolic Diseases, Children’s Hospital Affiliated to Zhengzhou University, Zhengzhou University, Zhengzhou, Henan, China; ^2^ Riphah Institute of Pharmaceutical Sciences, Riphah International University, Islamabad, Pakistan; ^3^ Department of Pharmacy, IQRA University Islamabad Campus (Chak Shahzad), Islamabad, Pakistan; ^4^ Department of Pharmaceutical Technology, Institute of Pharmacy, University of Innsbruck, Innsbruck, Austria; ^5^ Department of Neurosurgery, University of Florida, Gainesville, FL, United States; ^6^ Institute of Translational Medicine, Zhejiang Shuren University, Hangzhou, China

**Keywords:** ethnomedicine, diabetes, treatment, clinical trail, outcome, systematic review

## Abstract

Diabetes mellitus (DM) is a widespread metabolic disorder with a yearly 6.7 million deaths worldwide. Several treatment options are available but with common side effects like weight gain, cardiovascular diseases, neurotoxicity, hepatotoxicity, and nephrotoxicity. Therefore, ethnomedicine is gaining the interest of researchers in the treatment of DM. Ethnomedicine works by preventing intestinal absorption and hepatic production of glucose as well as enhancing glucose uptake in muscles and fatty tissues and increasing insulin secretion. A variety of plants have entered clinical trials but very few have gained approval for use. This current study provides an evaluation of such clinical trials. For this purpose, an extensive literature review was performed from a database using keywords like “ethnomedicine diabetes clinical trial”, “clinical trials”, “clinical trial in diabetes”, “diabetes”, “natural products in diabetes”, “ethno-pharmacological relevance of natural products in diabetes”, etc. Clinical trials of 20 plants and natural products were evaluated based on eligibility criteria. Major limitations associated with these clinical trials were a lack of patient compliance, dose-response relationship, and an evaluation of biomarkers with a small sample size and treatment duration. Measures in terms of strict regulations can be considered to achieve quality clinical trials. A specific goal of this systematic review is to discuss DM treatment through ethnomedicine based on recent clinical trials of the past 7 years.

## Introduction

Diabetes mellitus (DM) is a healthcare challenge prevailing worldwide with 8.75 million individuals affected with type 1 diabetes mellitus (T1DM) alone (using the Markov Model and machine learning techniques) with 1.52 million being children less than 20 years of age. 182,000 deaths were estimated worldwide due to T1DM in 2022 even after allopathic treatment (International Diabetes Federation ATLAS Report, 2022). An overall estimate made by IDF (International Diabetes Federation) in 2021 shows that 537 million adults are living with both types of DM [T1DM and type 2 diabetes (T2DM)], and it is expected to increase to 578 million by 2030 and 700 million by 2045 (International Diabetes Federation ATLAS, 2023).

With such a huge number of individuals affected with DM and its prevalence exacerbating worldwide, a proper treatment plan is the need of the hour. This has drawn the attention of researchers toward natural product strategies, focusing on the appropriate cure for DM. This shift in focus is attributed to complications associated with DM as well as the use of allopathic medicines. For instance, prolonged uncontrolled DM leads to complications such as amputation, vision loss, renal failure, and cardiovascular diseases (Rangaswami et al., 2020; Bhatti et al., 2021; Borderie et al., 2023). According to the World Health Organization (WHO) in 2022, 1 million individuals suffer from diabetes-induced blindness. Patients suffering from DM have a threefold increased risk of stroke and renal failure (World Health Organization, 2023). Furthermore, oral hypoglycemic drugs like biguanides, thiazolidinediones, sulfonylureas, and *a*-glucosidase inhibitors though play a vital role in DM management but cause significant adverse effects such as hepatorenal disturbances, hypoglycemic coma, *etc.* (Menne et al., 2019; Joseph, 2021). Therefore, there is a rising concern in ethnomedicine (*ethno* means ethnicity, cultural group, or people, and *ethnomedicine* means the study of healing practices or medical systems utilizing traditional medicine and theoretical perspectives of culture (Erickson, 2016)) due to its safety compared to allopathic or chemical anti-diabetic agents (Tran et al., 2020). As a matter of fact, plants have always been a source of medicine and many anti-diabetic agents extracted from plants have received FDA approval, for example, metformin derived from *Galega officinalis* (Asif et al., 2020).

Several natural products have extensively been used in the cure of DM in the context of pre-clinical and clinical studies (Abdelmagyd et al., 2019; Lu et al., 2019; Venkatakrishnan et al., 2019; Rahman et al., 2022). Outcomes from these clinical trials provide evidence for ethnomedicine in the cure of DM. For instance, zinc and copper have been studied to regulate insulin receptors causing extended insulin activity (Bjørklund et al., 2020), and magnesium supplementation showed beneficial effects on blood pressure in T2DM patients (Asbaghi et al., 2021). Cinnamomum showed beneficial effects in the reduction of the serum levels of glucose (Namazi et al., 2019) and clove (*Syzygium aromaticum*) possesses an enhanced antioxidant capacity in patients with diabetes (Thomas et al., 2022). Olive oil and garlic (*Allium sativum*) are useful in preventing dyslipidemia in T2DM patients (Memon et al., 2018), and garlic tablets allicor is useful in maintaining plasma lipid profile in T2DM patients (Sobenin et al., 2008), and so on. Likewise, various plants with distinctive effects such as fenugreek (*Trigonella foenum-graecum*) used in controlling blood glucose (A. AHMAD et al., 2020), bitter melon (*Momordica charantia*) used in enhancing insulin sensitivity, repairing damaged pancreas islet β-cells, stimulating insulin secretion (Gao et al., 2021), and lowering blood glucose (Mahwish et al., 2021), star anise (*Illcium Verum Hook. F*) known for its anti-oxidant properties in DM (Dewajanthi et al., 2020) and multiple others have been studied but are not approved for clinical studies in DM. 

Various clinical trials regarding the use of ethnomedicine in DM have been conducted and published. Post-prandial and fasting blood glucose levels, insulin sensitivity, HbAlc, glycemic exposure and variability, and hypo- and hyperglycemia were amongst the few parameters that were measured in these trials to assess the effects of natural products in improving the quality of life (R. Ahmad et al., 2021) and general health of the patients (Fagherazzi and Ravaud, 2019). This study is designed to collect, analyze, and document primary ethnomedicine for DM and provide an update on the current understanding of ethnopharmacological studies for DM. An elucidation of this will facilitate the identification of plant species/natural products that can be used for the effective treatment of DM patients. The clinical trials discussed in this systematic review were primarily based on studies from 2015 to 2023.

## Methods

A systemic review of interventional and observational studies was conducted that highlighted the efficacy and safety facts of clinical trials conducted on ethnomedicine for diabetes. This included qualitative and quantitative studies which help in enriching the knowledge based on the impacts of ethnomedicine on DM patients and in developing possibilities for ethnomedicine in complementing clinical practices.

### Eligibility criteria

The eligibility criteria presented in Table 1 were developed according to the qualification standards of the PICO (Population, Intervention, Comparison, and Outcome) approach adhering to PRISMA (Preferred Reporting Items for Systematic Reviews and Meta-Analyses) rules. Briefly, the current review includes only those studies that fulfilled any parameter of the eligibility criteria: plants that are believed to be anti-diabetic, assessing outcome measures related to DM such as increasing insulin sensitivity or lowering blood glucose levels among patients/participants in clinical studies, and clinical studies that have been completed (Table A1).

**TABLE 1:** Research questions and eligibility and exclusion criteria for systemic review based on clinical studies on ethnomedicines for Diabetes Mellitus.

Research questions	Eligibility/Inclusion criteria		Exclusion criteria
	General	Particular	
1. Does ethnomedicine provide a safe and effective treatment for DM patients?	1. Research articles. Peer-reviewed articles only based on clinical studies that defined and tested the safety and efficacy of ethnomedicine in all types of DM.	1. Clinical studies that clearly stated the aims/objectives of conducting clinical studies on ethnomedicine for DM.	1. Repeated/duplicate articles for clinical studies were excluded
2. Is ethnomedicine effective in the management of diabetic complications and in correcting metabolic abnormalities through a variety of mechanisms?	2. Research articles based on clinical studies with the title and/or abstract containing ethnomedicine of DM as related keywords	2. Clinical studies that clearly stated/mentioned the parameters that were assessed during clinical studies	2. Clinical studies that were not published/peer-reviewed were not included to ensure the high quality of available data
3. Does ethnomedicine decrease DM prevalence?	3. Research articles based on clinical studies in which any of the research questions were addressed	3. Clinical studies that showed the impact of observed results in managing diabetic complications in DM patients and/or clearly mentioned the endpoints of the study	3. Clinical studies that were published in languages other than English were not considered
4. What are the parts of bioactive metabolites/plants that contribute to the effectiveness in treating DM?	4. Articles based on clinical studies proving to have relevant content, conclusion, and/or scientific knowledge that might be deduced from it	4. Clinical studies that indicated the possible impact of ethnomedicine on the quality of life and life span of patients with DM.	4. Reviews (short/mini-reviews, narrative reviews, abstracts from conferences, letters, short communications, PhD theses, and comments) were not accepted
5. Can ethnomedicine for DM complement and not replace the existing/conventional anti-diabetic agents/therapies?	5. Randomized, multicenter, open-labeled, global clinical trials irrespective of whether the studies were conducted on humans	5. Clinical studies that described reasons for the growing need for investigation on ethnomedicine for DM.	5. Clinical studies conducted on patients with DM that were not assessing the effect of ethnomedicine on diabetes
6. Is ethnomedicine valuable in improving patients’ quality of life and increasing the life span of patients with DM and/or better responds to the needs of patients?	6. Research articles mentioning the completion of clinical studies	6. Clinical studies that identified prerequisites/necessary conditions required for the implementation of ethnomedicine in clinical settings	6. Natural products with established applications/properties in DM that have not gained approval for clinical studies as well as studies conducted on animals
7. Do clinical studies/trials encourage/promote appropriate clinical approaches to achieve expected results?	7. Research articles with clinical studies including all age groups and/or whole populations as well as subgroups with single or combination treatments	7. Clinical studies emphasizing hurdles/challenges in the implementation of ethnomedicine for DM in clinical settings	7. Phase 0 clinical studies and those evaluating minerals, vitamins, and/or conventional therapies/anti-diabetic agents

Abbreviations used: DM-Diabetes Mellitus, PhD-Doctor of Philosophy.

### Population and setting

The study population comprised all types of patients with DM (T1DM, T2DM, gestational diabetes, and juvenile diabetes) in any geographical area and animals involved in clinical studies for ethnomedicine for DM.

### Intervention

Clinical studies pertaining to assessing the effect/impact of ethnomedicine on different parameters in DM, evaluating the efficacy, safety, and clinical implementations and/or large-scale applications of clinical studies from 2015 to 2023.

### Ethical review

Ethical approval was not mandatory as this review did not include any animal or human subjects.

### Outcome measures

This systemic review was written to assess whether ethnomedicines for DM have an impact on the quality of life of all types of patients with DM. To carry out this review, included clinical studies were assessed for at least one of the parameters viz*:* i) its provision of safe and effective treatment for patients with DM, ii) its effectiveness in the management of diabetic complications and alleviation of metabolic abnormalities *via* multiple mechanisms, iii) decreased DM prevalence, iv) parts of bioactive metabolites/phytoconstituents/plants contributing to the effectiveness in treating DM, v) its effectiveness in complementing conventional anti-diabetic medicines, vi) its significance in enhancing the quality of life and increasing the life span of patients with DM, and vii) its ability to promote/encourage appropriate strategies in achieving expected results.

### Database and search strategy

**FIGURE 1:**
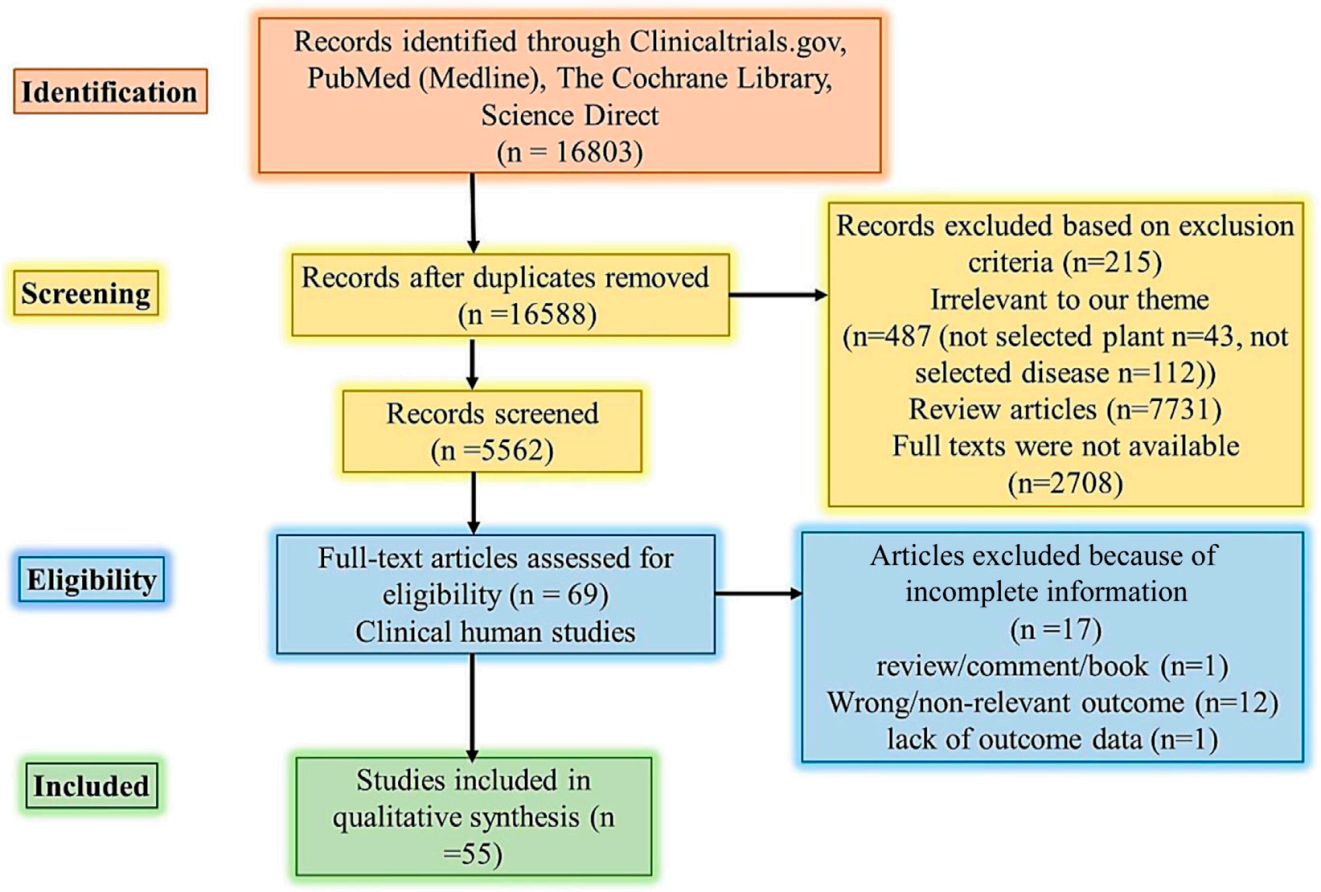
PRISMA flow diagram literature search and selection. Abbreviations: PICO Population, Intervention, Comparison, and Outcome.

The method used followed the PICO approach (Figure 1). The hunt for articles on clinical studies published in English was conducted through online databases (PubMed or Medline, Cochrane, ScienceDirect, and Clinicaltrials.gov). The following keywords were used and retained in query databases: ethnomedicine diabetes clinical trial, ethnomedicine diabetes randomized clinical trial, *Aloe vera*, diabetes clinical trial, Aloe barbadensis, Aloe chinensis, Aloe elongate, American ginseng, *Panax quinquefolius,* Bilberry, *Vaccinium myrtillus,* Cinnamon, diabetes *Cinnamomum aromaticum,* Fenugreek, *Trigonella foenum-graecum*, Garlic, Gymnema, diabetes Jambolan seeds, Bitter melon, Maitake, Neem, Nopal, Onion, Psyllium, Siberian Turmeric (Table A2).

### Study selection and data extraction

The selection and addition of research articles were done stepwise. Firstly, titles and abstracts were scrutinized for eligibility to avoid the addition of duplicate articles. Later, the full text of articles that were found relevant after scrutiny were retrieved and checked against the checklist/eligibility criteria (Table 1). Information mined was the year of study, country/continent/geographical area, the year of publication, clinical study design, intervention, aims of studies, and outcome measures. Data mining was done by two authors (GRE and KS).

### Quality *versus* risk of bias assessment in clinical studies

Two authors (KS and GRE) executed a methodological quality assessment based on the ROB (Cochrane Collaboration Risk of Bias) tool and the Newcastle Ottawa scale (NOS) for clinical studies. Negative bias impacts on clinical studies, and to prevent this, methodologies were placed within the research. Quality assessment relates to the inclusion of methodological quality within research, while ROB relates to the implication of such methodology for study results (Furuya-Kanamori et al., 2021). The ROB tools comprise seven domains, i.e., i) sequence creation, ii) allocation concealment, iii) blinding, iv) imperfect outcome measure data, v) selective outcome reporting, and vi) other sources of bias. The NOS comprises i) the basis of selection of clinical study, ii) comparability of groups, iii) ascertainment of exposure, and iv) evaluation of outcome measures. The quality of this study was rated with scores from zero (lowest score/grade) to nine (highest score/grade) and those studies with the lowest/low operational quality were excluded in order to avoid misleading outcomes (Côté et al., 2020).

## Discussion

DM is a life-threatening disease throughout the world caused by metabolic disorders mainly due to high blood glucose because of insulin inadequate synthesis by the pancreas or insufficient insulin activity. In addition to high blood glucose levels, the abnormal insulin level also alters lipid metabolism thereby raising the risk of vascular diseases (Nugroho et al., 2022). The major types of DM are T1DM, T2DM, and gestational diabetes. Being a global threat, 463 million people suffered from DM in 2019, that was 9.3%, and the prevalence can reach 10.2% in 2030% and 10.9% in 2045. This is because the prevalence of the prediabetes stage is 20.2% globally, which can lead to numerous diabetic complications involving neuropathy, nephropathy, and retinopathy (Fareed et al., 2017; Saeedi et al., 2019).

Several chemical drugs presently utilized as first-line treatment options that are administered orally to control the blood glucose level include thiazolidinediones (pioglitazone), biguanide (metformin), sodium-glucose cotransporter inhibitor (empagliflozin), GLP-1 agonists (exenatide, dulaglutide), dipeptidyl peptidase 4 inhibitors (sitagliptin, vildagliptin), meglitinides (nateglinide and repaglinide), and sulfonylureas (glipizide, glyburide) (Chaudhury et al., 2017; Kalsi et al., 2017). These drugs are linked with several side effects like weight gain, hepatotoxicity, nephrotoxicity, and risk of cardiovascular illnesses (But et al., 2014; Krass et al., 2017). Therefore, plant-based therapy is recommended as an alternative treatment option due to comparable safety and sustainability. Over 1,200 plants are used for the traditional treatment of diabetes and are preferred to chemical agents due to their lesser side effects and economic burden (Asgary et al., 2016; Ullah et al., 2019). Flavonoids, alkaloids, phenolics, and tannins are the most common active ingredients in plants used for diabetes treatment (Mamun et al., 2014). Hypoglycemic plants work through several mechanisms including the prevention of glucose absorption and production in intestines and liver cells, respectively, enhancing glucose absorption into muscle and fat tissues and increasing insulin secretion (Hegazy et al., 2013).

Literature reviews showed that the majority of plants have proven hypoglycemic effects in animal models. Afterward, their clinical trials had also been conducted. We have compiled the recent clinical trials of plants reported in the last 7 years from 2015 to 2023. During clinical trials, the extracts of antidiabetic plants were chiefly administered orally in combination with normal lifestyle modifications and/or other antidiabetic and anti-hyperlipidemic drugs for a specified treatment period compared to the placebo-controlled groups. The patient’s lifestyle and other parameters remained constant during the treatment period. The main parameters evaluated for hypoglycemic efficacy in all clinical trials were HbA1c and fasting blood and 2 hours postprandial blood sugar. The risk parameters for cardiovascular complications were serum lipids like cholesterol and triglyceride, and biomarkers of vascular inflammation, endothelial function, and oxidative stress. As shown in Tables 2, 3, plants had considerable hypoglycemic efficacy in clinical trials with safety and minimized risk of cardiovascular complications *via* hypoglycemic, hypolipidemic, and antioxidant properties.

**TABLE 2:** Details regarding clinical trials of plants in diabetes patients.

Name	Botanical name	Administered form	Participants	Intervention	Treatment period	Results	References
Aloe	*Aloe Vera*	300 mg dry gel capsule	240	Group A: Metformin + Glimepiride with rosuvastatin and 300 mg Aloe vera capsule twice a day as add-on therapy	84 days	Reduced blood sugar, triglyceride, and cholesterol in group A	Shoaib et al. (2022)
				Group B: Metformin + Glimepiride with rosuvastatin			
American ginseng	*Panax quinquefolius*	Extract capsules	38	One capsule three times per day (3 g/day) 40 min pre-prandial with lifestyle modification and pharmacotherapy. Corn starch (3 g/day) was given as control	8 weeks	↓ HbA1c and fasting blood glucose due to increased insulin indicated in serum	Vuksan et al. (2019)
						↓ blood lipids and systolic blood pressure and increased nitric oxide generation due to improved endothelial function	
Coadministration of American ginseng and konjac-based fiber	*Panax quinquefolius* and konjac-based fiber blend	Dried AG roots	39	Interventions comprised 6 g/day of fiber from KGB and 3 g/day of AG in addition to medications, diet, and lifestyle modification. Corn starch (3 g/day) was given as control instead of AG.	12 weeks	Significantly reduced both HbA1c and circulating lipid concentrations	Jenkins et al. (2018)
Coadministration of Korean Red Ginseng and American ginseng	*Panax ginseng* and *Panax quinquefolius*	Dried AG roots	80	Patients were given AG and Rg3-KRG of 2.25 g/day while wheat-bran was used as control	12 weeks	Greater reduction in HbA1c and serum lipids with no toxicity concerns	Jovanovski et al. (2021)
Bilberry	*Vaccinium myrtillus*	Bilberry fruit	47	Group A received bilberry purée of 200 g/day and dried bilberries of 40 g/day	8 weeks	Fasting serum hippuric acid was considerably raised only in group A, inducing reduced fasting plasma glucose concentration and raised insulin secretion	DeMello et al. (2017)
				Group B was given fresh strawberries, raspberries, and cloudberries, each of 100 g in their daily diet.			
				The control group continued with a berry-restricted diet.			
Bitter melon	*Momordica charantia*	Capsules of 500 mg containing dried powder of fruit pulp	24	Capsule of 500 mg was administered four times a day	3 months	Reduced glucose AUC, HbA1c, 2-h glucose, weight, BMI, and fat percentage, with an increase of insulin AUC and total insulin discharge	Cortez et al. (2018)
				Calcined magnesia was given as a placebo			
Cinnamon	*Cinnamomum cassia*	Stem bark supplement capsule	99	All participants randomly received either a 500 mg capsule of cinnamon or a placebo capsule daily	60 days	Cinnamon reduced plasma glucose, HbA1c, triglyceride, and BP and increased HDL-C and eGFR in T2DM patients	Sengsuk et al. (2016)
Ceylon cinnamon	*Cinnamomum zeylanicum*	Stem bark supplement capsule	30	Each subject was given 85 mg, 250 mg, and 500 mg per day in the 1st, 2nd, and 3rd months, respectively	3 months	Demonstrated anti-hyperlipidemic and blood pressure lowering effects in healthy adults	Ranasinghe et al. (2017a)
						No significant hepatotoxicity and anti-coagulation toxicity were observed	
Fenugreek	*Trigonella foenum-graecum*	Seed extract (Fenfuro)	154	Fenfuro (500 mg) was used twice daily	90 days	A decline in fasting sugar, HbA1c, and post-prandial sugar levels	Verma et al. (2016)
						Reduced dosage of anti-diabetic treatment was reported in 48.8% of patients	
Gymnema	*Gymnema sylvestre*	Powder leaves in capsules	30	The treatment group received 300 mg of Gymnema b.i.d. Before a meal, and the control group was given a homologated placebo	12 weeks	Decreased 2-h OGTT, HbA1c, and serum lipids.Reduced dosage of anti-diabetic treatment was reported in 48.8% of patients	Gaytán et al. (2021)
						Reduced dosage of anti-diabetic treatment was reported in 48.8% of patients	
						Increased insulin sensitivity	
Garlic	*Allium sativum*	Aged Garlic Extract	26	Garlic extract or placebo was given as 1,200 mg/day for 4 weeks with 4 weeks washout period	4 weeks	Vascular inflammation, endothelial function, oxidative stress, and insulin resistance were not improved significantly	Atkin et al. (2016)
Ginger	*Zingiber officinale*	Root powder	45	Ginger powder (2 g) and wheat flour (2 g) were given to intervention and placebo groups, respectively	10 weeks	T2DM patients had a significant decrement in ADMA serum concentration and there was a small sICAM-1 reduction in the treatment group	Zarezadeh et al. (2019)
Jamun	*Syzygium cumini*	Seed powder	99	The treatment group was given jamun seed powder of 5 g/day twice daily before a meal, and the control group was given placebo powder	90 days	Significantly reduced HbA1c, fasting plasma, and post-prandial plasma glucose were observed in the treatment group	544
Psyllium	*Plantago ovata*	Psyllium seed husk	51	Patients received 10 g of psyllium pre-mixed in cookies twice per day or placebo cookies	12 weeks	Decreased body weight, HbA1c, and fasting plasma glucose were observed	Noureddin et al. (2018)
						Also, there were reduced cholesterol, triglycerides, and constipation symptoms in T2DM patients	
Turmeric	*Curcuma longa*	Rhizome powder capsule	80	The treatment group received 700 mg capsules thrice a day after meals and cornstarch flour was provided to the placebo group	8 weeks	Reduced body weight and lipids in hyperlipidemic patients with T2DM.	Adab et al. (2019)

**TABLE 3:** Details regarding clinical trials of combined herbal products in diabetes patients.

Combined product	Plants	Administered form	Participants	Intervention	Treatment period	Results	References
Combined herbal capsule	*Galega officinalis* leaves, *Cinnamomum zeylanicum, Trigonella foenum-graecum* seeds, *Berberis vulgaris* fruit, and *Vaccinium bracteatum* thumb leaves	500 mg capsule	80	Combined herbal and placebo capsules were administered twice a day before a meal to intervention and placebo groups, respectively	3 months	HbA1c, fasting blood sugar, and 2 hours postprandial were reduced significantly	Ghafarzadegan et al. (2021)
						Also, there were decreased serum lipids in patients with T2DM.	
Plantabetics capsule of fenugreek, Indian gooseberry, turmeric, and grape seed	*Curcuma longa, Trigonella foenum-graecum, Emblica officinalis,* and *Vitis vinifera*	350 mg capsule	50	One capsule two times daily was given alongside their diabetes medication	84 days	Reduced HbA1c and postprandial blood and fasting blood sugar	Banerji and Banerjee (2016)
Viabet capsule	*Cinnamomum cassia, Urtica dioica, Trigonella foenum-graecum, Stevia rebaudiana,* and *Cyamopsis tetragonoloba*	500 mg capsule	30	Viabet capsules of 500 mg and a placebo were given randomly to participants thrice a day	3 months	HbA1C and body weight reduced significantly in the treatment group with no effects on fasting blood sugar	Tanzidi et al. (2023)

## Limitations and future prospective

Despite the good pharmacological effects of plants, several limitations were observed in clinical trials in terms of the heterogeneity of the study population, clinical protocols, and design and outcome evaluation parameters. Regarding the small sample size, an important consideration was that the number of patients initiating treatment was higher than those completing treatment which may be attributable to compliance and financial issues. The recruitment of healthy people for a clinical trial always remains a difficult task for researchers. Furthermore, the short study duration was a common limitation in all clinical trials (Zarrintan et al., 2016; Ranasinghe et al., 2017a; Asadi et al., 2019; Soltanian and Janghorbani, 2019; Gaytán Martínez et al., 2021). However, population size and duration of treatment must be considered because a larger study population with a longer treatment duration will depict more reliable safety and efficacy results. Another important limitation was the burden of polypharmacy and compliance of patients that were affected due to large size dosage form, i.e., capsule or tablet, with frequent dosing each day (Talaei et al., 2017; Tavakoly et al., 2018; Jovanovski et al., 2021). Moreover, clinical trials did not portray the dose-response relationship in patients, instead, even phase 1 clinical trials were based on single-dose utilization that cannot depict a complete safety profile (Sengsuk et al., 2016; Asadi et al., 2019; Jovanovski et al., 2021). The inclusion criteria were a crucial reason why some clinical trials failed to produce effective glycemic control. The inclusion criteria must specify that patients with diabetes with a history of a number of years with increased HbA1c levels can enter the study and/or set the exclusion criteria for patients with diabetes with high HbA1c levels or having diabetes for a long period of time (Zarrintan et al., 2016; Zarvandi et al., 2017). A lack of control groups was observed in some clinical trials which must not be ignored before drawing conclusions (Banerji and Banerjee, 2016; Ranasinghe et al., 2017b). Also, none of the clinical trials addressed if there was a difference between male and female patients with diabetes. Most of the population in the studies were women which may be due to a low willingness of men to participate in the study.

## Conclusion

Evaluating biomarkers for therapeutic and toxicological effects can support positive outcomes of the clinical trials being conducted. Most importantly, before initiating a clinical trial, the quantity and quality of plant phytochemicals must be verified to prevent differences due to geographical origins and environmental factors.

## Data Availability

The original contributions presented in the study are included in the article/supplementary material, further inquiries can be directed to the corresponding author.

## Appendix

**TABLE A1:** Search strategy according to the PICO guidelines.

PICO category	Reasons	Counts
P	Wrong setting/sample size	0
I	Wrong procedure/Insufficient intervention data	0
C	review/comment/book	1
O	Wrong/non-relevant outcome lack of outcome data	12
		1
Not a PICO-based exclusion criterion	Irrelevant to our theme (not selected plant or not selected disease)	487 (43 112)
	Full texts were not available	7,731
		2,708

**TABLE A2:** Search strategy of literature on various databases.

Database	Keywords
Science Direct	“ethnomedicine diabetes clinical trial” [Title/Abstract] AND “ethnomedicine diabetes Randomized clinical trial” [Title/Abstract] AND “*Aloe vera* diabetes clinical trial” [Title/Abstract] AND “diabetes clinical trial Aloe barbadensis” [Title/Abstract] AND “diabetes clinical trial Aloe chinensis” [Title/Abstract] AND “diabetes clinical trial Aloe elongate” [Title/Abstract] AND “diabetes clinical trial American ginseng” [Title/Abstract] AND “diabetes clinical trial *Panax quinquefolius*” [Title/Abstract] AND “diabetes clinical trial Bilberry” [Title/Abstract] AND “diabetes clinical tiral *Vaccinium myrtillus*” [Title/Abstract] AND “diabetes clinical trial Cinnamon” [Title/Abstract] AND “diabetes clinical trial *Cinnamomum aromaticum*” [Title/Abstract] AND “diabetes clinical trial Fenugreek” [Title/Abstract] AND “diabetes clinical trial *Trigonella foenum-graecum*” [Title/Abstract] AND “diabetes clinical trial Garlic [Title/Abstract] AND “diabetes clinical trial Gymnema” [Title/Abstract] AND “diabetes clinical trial Jambolan seeds” [Title/Abstract] AND “diabetes clinical trial Bitter melon” [Title/Abstract] AND “diabetes clinical trial Maitake” [Title/Abstract] AND “diabetes clinical trial Neem” [Title/Abstract] AND “diabetes clinical trial Nopal” [Title/Abstract] AND “diabetes clinical trial Onion” [Title/Abstract] AND “diabetes clinical trial Psyllium” [Title/Abstract] AND “diabetes clinical trial Siberian Turmeric”
PubMed	“ethnomedicine diabetes clinical trial” [Title/Abstract] AND “ethnomedicine diabetes Randomized clinical trial” [Title/Abstract] AND “*Aloe vera* diabetes clinical trial” [Title/Abstract] AND “diabetes clinical trial American ginseng” [Title/Abstract] AND “diabetes clinical trial *Panax quinquefolius*” [Title/Abstract] AND “diabetes clinical trial Bilberry” [Title/Abstract] AND “diabetes clinical trial *Vaccinium myrtillus*” [Title/Abstract] AND “diabetes clinical trial Cinnamon” [Title/Abstract] AND “diabetes clinical trial *Cinnamomum aromaticum*” [Title/Abstract] AND “diabetes clinical trial Fenugreek” [Title/Abstract] AND “diabetes clinical trial *Trigonella foenum-graecum*” [Title/Abstract] AND “diabetes clinical trial Garlic [Title/Abstract] AND “diabetes clinical trial Gymnema” [Title/Abstract] AND “diabetes clinical trial Jambolan seeds” [Title/Abstract] AND “diabetes clinical trial Bitter melon” [Title/Abstract] AND “diabetes clinical trial Maitake” [Title/Abstract] AND “diabetes clinical trial Neem” [Title/Abstract] AND “diabetes clinical trial Nopal” [Title/Abstract] AND “diabetes clinical trial Onion” [Title/Abstract] AND “diabetes clinical trial Psyllium” [Title/Abstract] AND “diabetes clinical trial Siberian Turmeric”
The Cochrane Library	“ethnomedicine diabetes clinical trial” [Title/Abstract] AND “ethnomedicine diabetes Randomized clinical trial” [Title/Abstract] AND “*Aloe vera* diabetes clinical trial” [Title/Abstract] AND “diabetes clinical trial American ginseng” [Title/Abstract] AND “diabetes clinical trial *Panax quinquefolius*” [Title/Abstract] AND “diabetes clinical trial Bilberry” [Title/Abstract] AND “diabetes clinical trial *Vaccinium myrtillus*” [Title/Abstract] AND “diabetes clinical trial Cinnamon” [Title/Abstract] AND “diabetes clinical trial *Cinnamomum aromaticum*” [Title/Abstract] AND “diabetes clinical trial Fenugreek” [Title/Abstract] AND “diabetes clinical trial *Trigonella foenum-graecum*” [Title/Abstract] AND “diabetes clinical trial Garlic [Title/Abstract] AND “diabetes clinical trial Gymnema” [Title/Abstract] AND “diabetes clinical trial Jambolan seeds” [Title/Abstract] AND “diabetes clinical trial Bitter melon” [Title/Abstract] AND “diabetes clinical trial Maitake” [Title/Abstract] AND “diabetes clinical trial Neem” [Title/Abstract] AND “diabetes clinical trial Nopal” [Title/Abstract] AND “diabetes clinical trial Onion” [Title/Abstract] AND “diabetes clinical trial Psyllium” [Title/Abstract] AND “diabetes clinical trial Siberian Turmeric”
Clinicaltrials.gov	“ethnomedicine diabetes clinical trial” [Title/Abstract] AND “ethnomedicine diabetes Randomized clinical trial” [Title/Abstract] AND “*Aloe vera* diabetes clinical trial” [Title/Abstract] AND “diabetes clinical trial American ginseng” [Title/Abstract] AND “diabetes clinical trial *Panax quinquefolius*” [Title/Abstract] AND “diabetes clinical trial Bilberry” [Title/Abstract] AND “diabetes clinical trial *Vaccinium myrtillus*” [Title/Abstract] AND “diabetes clinical trial Cinnamon” [Title/Abstract] AND “diabetes clinical trial *Cinnamomum aromaticum”* [Title/Abstract] AND “diabetes clinical trial Fenugreek” [Title/Abstract] AND “diabetes clinical trial *Trigonella foenumgraecum*” [Title/Abstract] AND “diabetes clinical trial Garlic [Title/Abstract] AND “diabetes clinical trial Gymnema” [Title/Abstract] AND “diabetes clinical trial Jambolan seeds” [Title/Abstract] AND “diabetes clinical trial Bitter melon” [Title/Abstract] AND “diabetes clinical trial Maitake” [Title/Abstract] AND “diabetes clinical trial Neem” [Title/Abstract] AND “diabetes clinical trial Nopal” [Title/Abstract] AND “diabetes clinical trial Onion” [Title/Abstract] AND “diabetes clinical trial Psyllium” [Title/Abstract] AND “diabetes clinical trial Siberian Turmeric”
